# Ablation of Siglec-E augments brain inflammation and ischemic injury

**DOI:** 10.1186/s12974-022-02556-1

**Published:** 2022-07-20

**Authors:** Lexiao Li, Yu Chen, Madison N. Sluter, Ruida Hou, Jiukuan Hao, Yin Wu, Guo-Yun Chen, Ying Yu, Jianxiong Jiang

**Affiliations:** 1 Department of Pharmaceutical Sciences, Drug Discovery Center, College of Pharmacy, University of Tennessee Health Science Center, Memphis, TN USA; 2 Department of Pharmacological and Pharmaceutical Sciences, College of Pharmacy, University of Houston, Houston, TX USA; 3 Children’s Foundation Research Institute at Le Bonheur Children’s Hospital, Department of Pediatrics, College of Medicine, University of Tennessee Health Science Center, Memphis, TN USA

**Keywords:** Cerebral ischemia, DAMPs, Infarct, Innate immunity, LPS, Microgliosis, Neuroprotection, OGD, PAMPs, Sialic acid

## Abstract

Sialic acid immunoglobulin-like lectin E (Siglec-E) is a subtype of pattern recognition receptors found on the surface of myeloid cells and functions as a key immunosuppressive checkpoint molecule. The engagement between Siglec-E and the ligand α_2,8_-linked disialyl glycans activates the immunoreceptor tyrosine-based inhibitory motif (ITIM) in its intracellular domain, mitigating the potential risk of autoimmunity amid innate immune attacks on parasites, bacteria, and carcinoma. Recent studies suggest that Siglec-E is also expressed in the CNS, particularly microglia, the brain-resident immune cells. However, the functions of Siglec-E in brain inflammation and injuries under many neurological conditions largely remain elusive. In this study, we first revealed an anti-inflammatory role for Siglec-E in lipopolysaccharide (LPS)-triggered microglial activation. We then found that Siglec-E was induced within the brain by systemic treatment with LPS in mice in a dose-dependent manner, while its ablation exacerbated hippocampal reactive microgliosis in LPS-treated animals. The genetic deficiency of Siglec-E also aggravated oxygen–glucose deprivation (OGD)-induced neuronal death in mouse primary cortical cultures containing both neurons and glial cells. Moreover, Siglec-E expression in ipsilateral brain tissues was substantially induced following middle cerebral artery occlusion (MCAO). Lastly, the neurological deficits and brain infarcts were augmented in Siglec-E knockout mice after moderate MCAO when compared to wild-type animals. Collectively, our findings suggest that the endogenous inducible Siglec-E plays crucial anti-inflammatory and neuroprotective roles following ischemic stroke, and thus might underlie an intrinsic mechanism of resolution of inflammation and self-repair in the brain.

## Introduction

Sialic acids are a group of monosaccharides with a nine-carbon backbone and are most commonly represented by *N*-acetylneuraminic acid (Neu5Ac) and *N*-glycolylneuraminic acid (Neu5Gc). The α_2,8_ or α_2,3_-linked oligopolymers or polymers by this subtype of monosaccharides construct the terminal decoration of the glycocalyx located on the cell surface. These sialoglycan structures serve as specific “identity code” ligands of host cells and are recognizable for corresponding membrane receptors of immune cells. This set of immunorecognition-involved receptors are referred to as sialic acid-binding immunoglobulin-type lectins or Siglecs. By far, 15 members of Siglecs have been identified in humans and nine in mice. They overall can be divided into two subfamilies: conserved Siglecs that can be found in different mammals and CD33-related Siglecs which do not have clear orthologs across species but rather functional paralogs (e.g., Siglec-9 and Siglec-E) [1]. The selective *trans-* and *cis-*binding between ligands and receptors leads to the activation of motifs in the intracellular domains, and the downstream signaling cascades are implicated in immunomodulation. Depending on those intracellular motifs, the immunomodulatory property of a specific Siglec can be inhibitory or stimulatory. Most Siglecs are inhibitory because they carry the immunoreceptor tyrosine-based inhibitory motif (ITIM) or ITIM-like domains. The others contain a positively charged amino acid residue in the transmembrane domain that enables their binding to DAP12 (also known as KARAP or TYROBP) that has an activating intracellular immunoreceptor tyrosine-based activation motif (ITAM) [2, 3]. As such, Siglecs regulate immune checkpoint mechanisms and are thought to mitigate the risk of autoimmunity amid innate immune attacks on parasites, bacteria, and carcinoma.

Siglec-E is one of the most representative members in mouse CD33-related Siglec subfamily. As a pattern recognition receptor located on the surface of myeloid cells, Siglec-E functions as a key immunosuppressive checkpoint molecule. The engagement between Siglec-E and the ligand α_2,8_-linked disialyl glycans activates the ITIM located in its intracellular domain. Since Siglec-E was first cloned about two decades ago [4], it has been extensively studied in a wide range of conditions, such as carcinoma [5–11], parasite infection [12–15], bacterial infection and sepsis [16–22], inflammatory lung diseases [23–25], chronic obstructive pulmonary disease [26], asthma [27, 28], atherosclerosis [29], hyperglycemia and diabetes [30, 31], and autoimmune diseases [32]. Siglec-E has also been widely found within the brain, particularly activated microglia [33, 34]; however, its functions in neuroimmune system remain largely unknown.

Accounting for about 5–15% of all cells in the CNS, microglia are widely recognized as the resident myeloid cells in the brain parenchyma and play essential roles in brain homeostasis and innate immunity [35]. As such, the reactive microgliosis is an intense reaction of microglia to inflammatory stimuli, such as exogenous pathogen-associated molecular patterns (PAMPs) and endogenous damage-associated molecular patterns (DAMPs), and is primarily featured by rapid, robust and sustained increases in activated microglia at the sites of brain insults. Given that microglia are the major cell type expressing Siglec-E within the brain, the functions of microglial Siglec-E have recently been studied in neuroinflammation-associated conditions. Activation of Siglec-E by microglia-derived polysialic acid ligands was shown to inhibit the inflammatory responses to lipopolysaccharide (LPS) stimulation in microglial cell line BV2 and to traumatic brain injury (TBI) in mice [34]. Likewise, interaction between microglial Siglec-E and sialic acids likely serves as a sensitive immune checkpoint axis and might underlie a molecular mechanism whereby anti-tumor immunity in glioma patients with steroid therapies is compromised [8]. To date, however, there is no study reported to investigate the role of microglial Siglec-E in brain inflammation and injury following ischemic stroke, a world-wide leading cause of death and adult disability.

Acute cerebral ischemia causes the primary brain injury mainly due to the malfunction of energy metabolism and neuronal excitotoxicity, while reactive microgliosis-associated neuroinflammatory processes induced by post-ischemic necrosis largely contribute to the delayed, secondary injury [36]. Understanding the molecular mechanisms underlying the post-stroke neuroinflammation and secondary injury is essential to the development of immunomodulatory therapies for ischemic stroke [37, 38]. In the present study, we investigated the effects of congenital ablation of Siglec-E in mouse primary microglia activated by LPS and in mouse cortical cultures subjected to oxygen–glucose deprivation (OGD). We also studied the roles of Siglec-E in the activation and morphological alterations of brain microglia in mice treated by LPS. Further, we examined the outcomes, such as neurological deficits, weight changes, and brain infarcts, in Siglec-E knockout mice after middle cerebral artery occlusion (MCAO). We propose that the elevated Siglec-E activity in microglia by ischemic injury alleviates the neuroinflammatory reactions and protects neurons from the subsequent escalated injuries, and thus might represent an intrinsic mechanism for inflammation resolution and brain tissue self-repair after cerebral ischemia.

## Methods

### Mouse primary microglial cultures

**Fig. 1:**
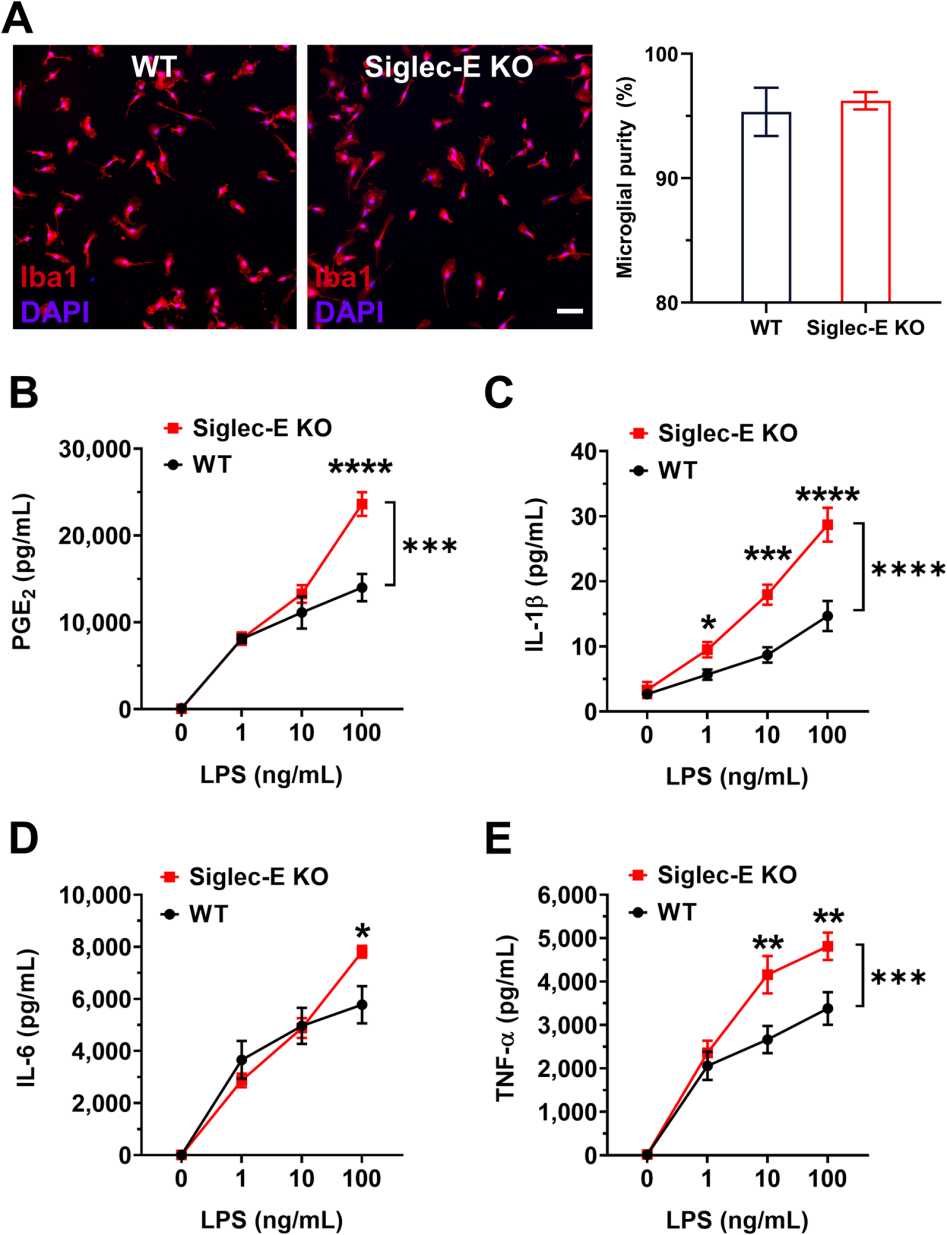
Siglec-E suppresses prototypical proinflammatory mediators in LPS-activated microglia. **A** Mouse primary brain microglia from C57BL/6 wild type and Siglec-E knockout pups were prepared in 48-well plates (4 × 10^4^ cells/well). The purity of microglia in these cultures was assessed by immunostaining for the microglial marker Iba-1 (red fluorescence) and compared (*n* = 9, *p* = 0.678, Mann–Whitney *U* test). Note that cell nuclei were stained with DAPI (blue fluorescence) to illustrate all cell types. Scale bar: 50 μm. The cultured cells were then stimulated with LPS (0, 1, 10, or 100 ng/mL) for 16 h. A number of key proinflammatory mediators that were secreted by LPS-activated microglia into the culture medium, such as PGE_2_ (**B**), IL-1β (**C**), IL-6 (**D**), and TNF-α (**E**), were measured by ELISA. Note that all these conventional proinflammatory mediators produced by microglia were induced by LPS treatment in a concentration-dependent manner and were further dramatically increased in the absence of Siglec-E (*n* = 8–12, *p < 0.05; **p < 0.01; ***p < 0.001; ****p < 0.0001, two-way ANOVA with post hoc Šidák multiple comparisons). All data are presented as mean ± SEM

The mouse primary microglial cultures were prepared as we previously described [39, 40]. Briefly, cortical tissues were isolated from newborn C57BL/6 mouse pups (P1) and were dissected in ice-cold Hank’s balanced salt solution (HBSS, Corning). Meninges and blood vessels were carefully removed. After repeated gentle trituration, filtration was performed to separate cells from tissue debris and chunks. The cells were then cultured in minimum essential medium (MEM) supplemented with 10% fetal bovine serum (FBS), 100 U/mL penicillin, and 100 µg/mL streptomycin in 0.001% poly-l-ornithine-coated flasks. Four to 5 days later, fresh complete MEM plus 2 ng/mL granulocyte–macrophage colony-stimulating factor (GM-CSF) was loaded (R&D Systems). After incubation for additional 4–5 days, matured microglia were collected by gentle agitation of the culture flasks. The harvested microglia were seeded into 48-well plates with fresh complete MEM plus 0.2 ng/mL GM-CSF (4 × 10^4^ cells/well). Such mouse primary cultures typically consisted of at least 95% Iba1-positive (Iba1^+^) cells as identified by immunocytochemistry (Fig. 1A). To stimulate microglia, these primary cultures were incubated with lipopolysaccharide (LPS) at concentrations ranging from 1 to 100 ng/mL overnight. ELISA was used to measure the conventional proinflammatory mediators in the cultural medium, including prostaglandin E2 (PGE_2_), interleukin 1β (IL-1β), IL-6, and tumor necrosis factor α (TNF-α). The manufacturers’ protocols in the ELISA kits were followed as we previously described [40].

### Mouse primary cortical cultures

Cortical cells were isolated from embryos (E18) of timed-pregnant C57BL6 mice as we previously described [41, 42]. Cells were seeded into poly-d-lysine (Sigma-Aldrich) coated 24-well plates (~ 300,000 cells/well) and cultured at 37 °C in a humidified incubator supplied with 5% CO_2_ and 95% air. Cells were cultured in neurobasal medium supplemented with B-27, sodium pyruvate, dextrose, l-glutamine, 100 U/mL penicillin, and 100 µg/mL streptomycin (Invitrogen). Half of the culture medium was replaced by fresh medium every 3–4 days, and immunocytochemistry confirmed that these cultures consisted of neurons, microglia, and astrocytes. On 10–14 days in vitro, these mouse primary neuron–glia mixed cortical cultures were subjected to oxygen–glucose deprivation (OGD) stress as we previously described [40, 43]. In brief, primary cells were incubated in glucose/glutamate-free DMEM (Gibco), and the culture plates were then sealed in a vacuum bag. The hypoxic condition was achieved through continuous aspiration by a vacuum pump which decreased the air pressure in the vacuum bag to 0.079 standard atmosphere and reduced the oxygen level to about 1.66% [40].

### Experimental animals

All animal experiments and procedures were approved by the Institutional Animal Care and Use Committee (IACUC) of the University of Tennessee Health Science Center and performed in line with the *Guide for the Care and Use of Laboratory Animals* (the *Guide*) from the National Institutes of Health (NIH). Mice were housed in standard humidity (45–50%) at room temperature (21–25 °C) under a 12-h light/dark cycle with ad libitum access to food and water. Siglec-E knockout mice were from the Mutant Mouse Regional Resource & Research Center (MMRRC_032571-UCD). Siglec-E gene in 129/Sv ES cells underwent targeted mutations, and the Siglec-E knockout mice were generated with the mutated 129/Sv ES cells. These mice were backcrossed to the C57BL/6 background for over eight generations to reduce the impact of 129-derived passenger gene mutations [17, 22, 44]. The wild-type littermates were used as controls for Siglec-E knockout mice.

### Mouse model of transient brain ischemia

Acute focal brain ischemia was prepared using middle cerebral artery occlusion (MCAO) as we previously described [40, 41, 45]. Buprenorphine SR-LAB (1.0 mg/kg, s.c.) for analgesia was given 1 h before the surgery. Mice were anesthetized via inhalation of vaporized isoflurane (Henry Schein) at 1.5–2%. The rectal temperature was monitored via a digital thermometer and was maintained at 37 °C using a heating pad. With an incision made on neck skin along the midline, soft tissues were gently separated to expose the vessel field on the right side. The vagal nerve was carefully dissociated from the common carotid artery, and the superior thyroid artery was ablated before the ligation of external carotid artery (ECA). The ECA was then cut off using a cauterizer (Bovie Medical Corporation). The occipital artery located above the CCA bifurcation was carefully ablated before the internal carotid artery (ICA) and pterygopalatine artery (PPA) became visible. The CCA and ICA were clamped with microvascular clips. With a 5-0 suture knot loosely prepared at the root of ECA, a tiny nick was made along the ECA stump for the insertion of the filament into ECA lumen. MCAO was achieved through delivering a 20-mm-long 6-0 silicon-coated Doccol filament into ICA by 10 mm to cease the blood supply into MCA. The properly placed filament was fixed by tightening the loose knot at the root of ECA. During the MCAO session, mice were placed above another heating pad for the restoration of consciousness. Thirty minutes later, reperfusion was started by withdrawing the Doccol filament. The researcher who performed this surgery had no knowledge about the genotype of mice. Successful MCAO surgery should result in marked neurological deficits. The assessment of post-stroke neurological deficits was performed also in a blinded manner as we previously described [40, 41]. The scoring scale was modified from Bederson’s and Longa’s versions as follows: 0, no deficit; 1, forelimb flexion; 2, reduced resistance to lateral push; 3, unidirectional circling; 4, barrel rolling/spinning; 5, no movement.

### Quantification of infarct and edema

Three days after reperfusion from MCAO, mice were euthanized under overdosed isoflurane anesthesia and subjected to transcardial perfusion with icy phosphate-buffered saline (PBS). A mouse coronal brain matrix was employed to help the preparation of 1-mm-thick coronal brain sections. These sections were stained with 0.2% TTC solution (2,3,5-triphenyltetrazolium chloride, Santa Cruz Biotechnology) for 20 min, aligned, and captured for digital imaging and analysis. The infarct size was quantified using ImageJ/Fiji software (NIH) in a blinded manner. The sum of infarct volume was performed following the stereological principle. The infarct volume was further adjusted based on the ipsilateral edema size in compliance with “Swanson’s correction”. The adjusted infarct volume was finally normalized to its contralateral brain volume in order to offset any artifactual impact including physical extension and shrinkage during the tissue processing.

### LPS model of brain inflammation

LPS from *Escherichia coli* O111:B4 was purchased from Sigma-Aldrich. For injection, LPS was dissolved in sterile isotonic saline and injected to mice with a volume of 10 mL/kg. Eight-weeks-old male C57BL/6 mice were weighed and randomized to treatments with LPS (3 or 5 mg/kg, i.p.) or saline [46]. Twenty-four hours after LPS injection, mice were anesthetized with isoflurane, then perfused with ice-cold PBS. Brain tissues were dissected and collected for biochemical and immunohistochemical analyses.

### Quantitative PCR

The total RNA from mouse brain tissues was extracted using the combination of TRIzol (Invitrogen), chloroform, and the PureLink RNA Mini Kit (Invitrogen). The purity and concentration of extractant were measured via the readings of A260/A280 ratio and A260, respectively, by a NanoDrop One microvolume spectrophotometer (Thermo Fisher). The single-stranded complementary DNA (cDNA) was synthesized using the SuperScript III First-Strand Synthesis SuperMix (Invitrogen) following the manual. The quantitative PCR (qPCR) was performed using 8 µl of 10 × diluted cDNA, 0.4 µM of primers and 2 × SYBR Green SuperMix (Bio-Rad Laboratories) with a final volume of 20 µl in a CFX96 Touch Real-Time PCR Detection System (Bio-Rad Laboratories). The cycling protocols were set as: 95 °C for 2 min followed by 40 cycles of 95 °C for 15 s and then 60 °C for 1 min. The fluorescent readings were set at the 60 °C step. Melting curve procedure was added to verify the uniformity of PCR product. The cycle of quantification for GAPDH gene was subtracted from the cycle of quantification measured for each gene of interest to yield ΔCq [40, 42]. Samples without cDNA template served as negative controls. The sequences of primers for qPCR were as follows: GAPDH, forward 5′-TGTCCGTCGTGGATCTGAC-3′ and reverse 5′-CCTGCTTCACCACCTTCTTG-3′; Siglec-E, forward 5′-GTCTCCACAGAGCAGTGCAACTTTATC-3′ and reverse 5′-TGGGATTCAACCAGGGGATTCTGAG-3′.

### Immunofluorescence staining

Immunostaining was performed as we previously reported [47, 48]. In brief, fixed coronal brain section (25 µm) underwent 60-min permeabilization and blocking with PBS containing 0.2% Triton X-100, 10% goat serum, and 22.52 mg/mL glycine. The sections were then incubated in rabbit anti-Iba1 polyclonal antibody (Wako cat. #019-19741, 1:200) and rat anti-Siglec-E monoclonal antibody (BioLegend cat. #677102, 1:100) at 4 °C overnight. Slices were then rinsed and incubated with anti-rabbit and anti-rat secondary antibodies conjugated with Alexa Fluor 546 or 488 (1:1000, Invitrogen) for 2 h. The slices were carefully mounted onto slides using the ProLong™ Gold antifade reagent (Invitrogen). The digital images were captured using a fluorescence microscope BZ-X800 (Keyence). The image processing and quantitative analyses were performed using the ImageJ/Fiji software (NIH).

### Statistical analysis

All statistical analyses were performed using GraphPad Prism or IBM SPSS Statistics. Datasets were first tested for outliers using the Grubb’s test, and then subjected to Shapiro–Wilk test of normality and Levene’s test of variance homogeneity. The Mann–Whitney *U* test and Kruskal–Wallis test were used for nonparametric tests, ANOVA were utilized for parametric tests, and *p* < 0.05 was considered statistically significant.

## Results

Siglec-E is abundantly expressed in mouse primary microglia, and its knockdown by shRNA transformed microglia to a state displaying aggravated proinflammatory characteristics when exposed to neural debris, mimicking DAMPs [33]. A similar relation between Siglec-E and reactive microgliosis following the exposure to PAMPs was also reported, as CRISPR/Cas9-mediated Siglec-E knockout in microglial cell line BV2 resulted in a stronger response to LPS stimulation [34]. However, investigation on congenital ablation of Siglec-E in primary microglia has not been reported to date. For that, we first cultured the mouse primary brain microglia derived from C57BL/6 wild type and Siglec-E knockout pups. Immunostaining for the microglial marker Iba-1 revealed that the purity of microglia was about 95% in the wild-type cultures and 96% in the Siglec-E knockout cultures (Fig. 1A). We then stimulated these cells with LPS (0, 1, 10, or 100 ng/mL). After overnight treatment, a number of key proinflammatory mediators secreted by LPS-activated microglia into the culture medium were measured by ELISA. It was found that LPS induced prostaglandin E2 (PGE_2_, Fig. 1B) and prototypical proinflammatory cytokines IL-1β (Fig. 1C), IL-6 (Fig. 1D), and TNF-α (Fig. 1E) in both wild type and Siglec-E knockout microglia in a concentration-dependent manner. However, the levels of PGE_2_ and cytokines were substantially higher in the Siglec-E knockout microglia when compared to the wild-type cells (Fig. 1B–E). These results demonstrate a key anti-inflammatory and immunosuppressive role for microglial Siglec-E in response to PAMPs like LPS.

**Fig. 2:**
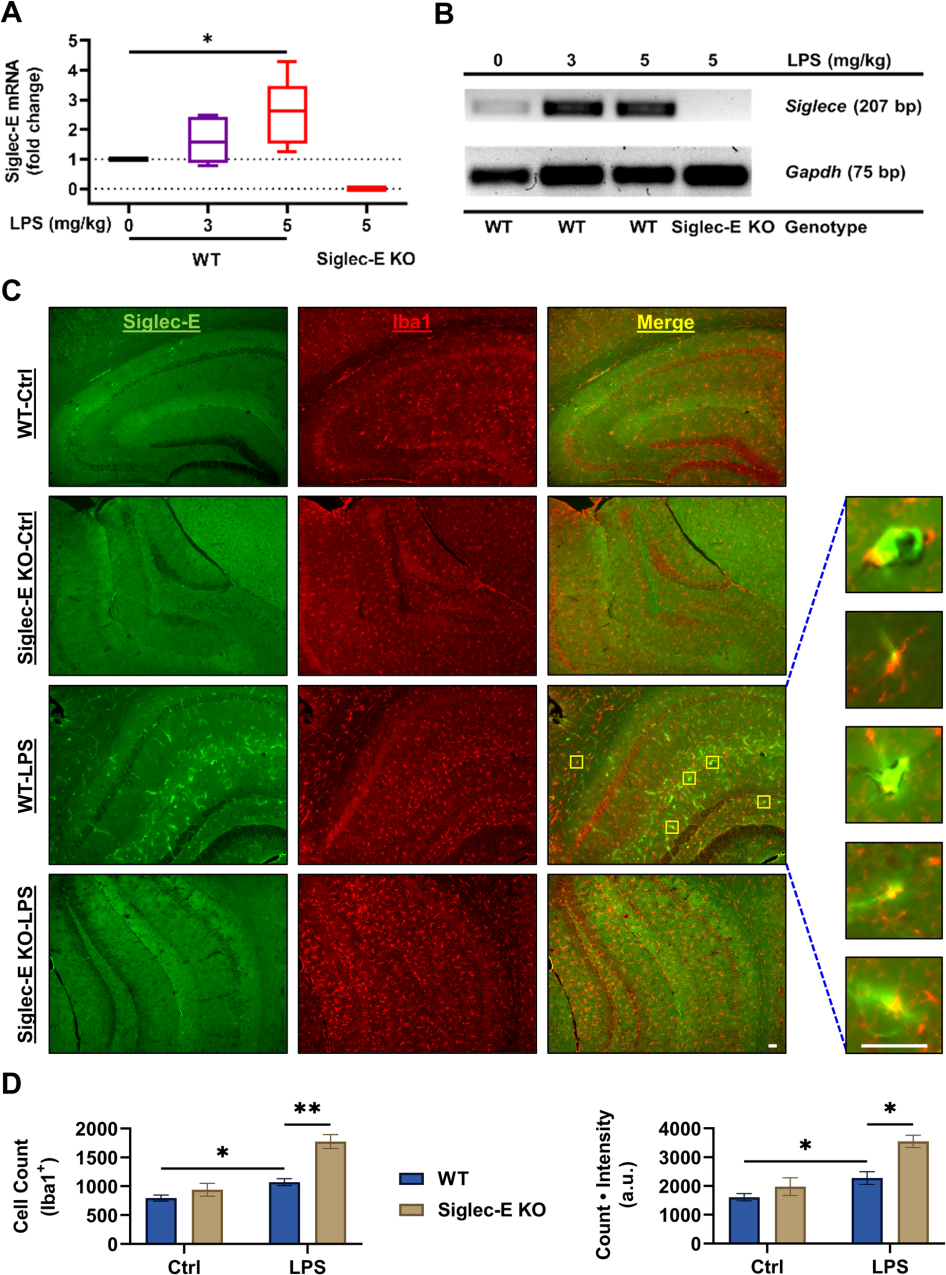
Ablation of Siglec-E exacerbates microglial activation in the brain. **A** C57BL/6 wild type and Siglec-E knockout mice were systemically treated by LPS (0, 3, or 5 mg/kg, i.p.), and 24 h later the mRNA expression of Siglec-E in the hippocampus was measured by qPCR (*n* = 5–8, *p = 0.01, Kruskal–Wallis test with post hoc Dunn’s multiple comparisons). Data are visualized using box plot. **B** Reverse transcription PCR was performed to examine the Siglec-E mRNA expression in the hippocampal tissues of wild type and Siglec-E knockout mice treated by LPS, with GAPDH as control. **C** Immunostaining for Siglec-E (green fluorescence) and Iba1 (red fluorescence) indicating the hippocampal microglial activation in wild type and Siglec-E knockout mice was performed 24 h after LPS treatment (5 mg/kg, i.p.). Representative images are presented here to exemplify the induction of Siglec-E and Iba1 as well as their colocalization in activated microglia. Scale bar: 50 μm. **D** Iba1-positive (Iba1^+^) microglia in the hippocampus were counted (Left) and their Iba1 expression levels were quantified by measuring the fluorescence intensity (Right) (n = 4–6, *p < 0.05, **p < 0.01, Mann–Whitney *U* test). Data are shown as mean ± SEM

The molecular mechanisms whereby the ablation of Siglec-E prevents the LPS-induced microglia-mediated inflammation is not fully understood. However, LPS induces inflammatory reactions via directly binding to the toll-like receptor 4 (TLR4), which is believed to be restrained by Siglec-E [44]. In the absence of Siglec-E, LPS-activated TLR4 is free to fully engage the downstream inflammatory mediators, although LPS may not directly act on Siglec-E. To further investigate the effects of LPS/TLR4-mediated inflammation on Siglec-E in vivo, we next examined the expression of Siglec-E in the brain after systemic treatment with LPS (3 or 5 mg/kg, i.p.) in adult C57BL/6 wild-type mice. It was found that LPS considerably induced mRNA expression of Siglec-E in the hippocampus in a dose-dependent manner, measured 24 h after the treatment (Fig. 2A). The induction of Siglec-E mRNA by LPS in wild-type mice and its absence in Siglec-E knockout mice were validated by reverse transcription PCR (Fig. 2B).

Interestingly, immunohistochemistry revealed a trend that, under basal conditions, the ablation of Siglec-E increased Iba1-positive (Iba1^+^) cells and the overall expression of Iba1 in the hippocampus (Fig. 2C), although the difference between the wild type and Siglec-E knockout mice was not statistically significant (Fig. 2D). The induction of Siglec-E by LPS in the brain tissues from wild-type mice was confirmed by immunohistochemistry, which also revealed considerable cellular colocalization between Siglec-E and microglial marker Iba1 in brain tissues from LPS-treated mice (Fig. 2C). Moreover, the LPS-induced reactive microgliosis featured by the increased Iba1^+^ cells and elevated expression of Iba1 in the brain was further enhanced in Siglec-E knockout mice when compared to the wild-type animals (Fig. 2D).

**Fig. 3:**
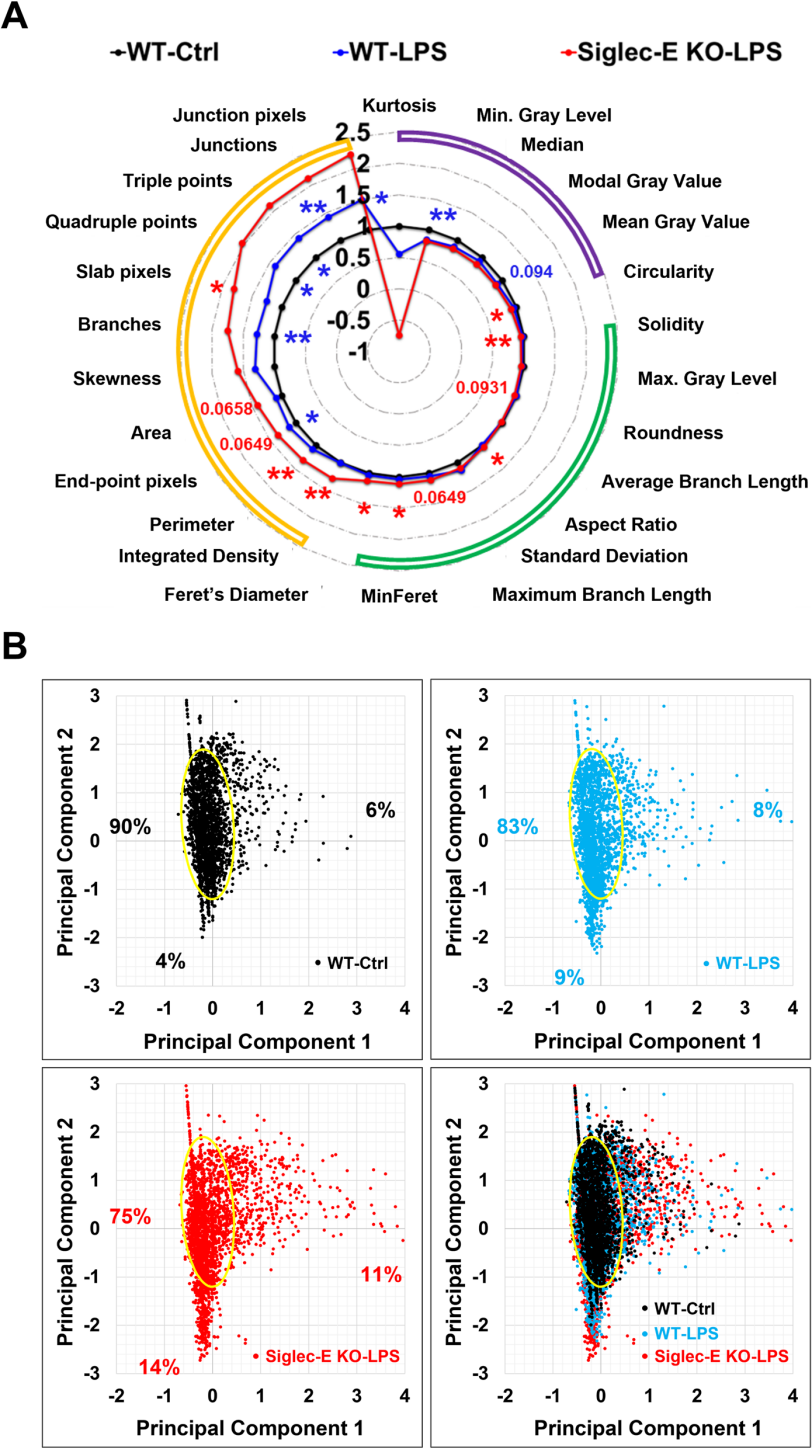
Siglec-E regulates the morphology of brain microglia. **A** The morphological analyses of brain microglia (Iba1^+^) in mice with 26 measurements (Table 1) were performed 24 h after LPS treatment (5 mg/kg, i.p.) using ImageJ/Fiji software. A radar chart was generated to show the morphological changes of brain microglia in mice by LPS treatment and deletion of Siglec-E. The statistical p values less than 0.1 were labeled (*n* = 4–6, *p < 0.05, **p < 0.01, Mann–Whitney *U* test). Note that the 26 measurements can be categorized into three clusters indicated by purple, green, and yellow arcs, showing that the measurements in wild-type control mice, when compared to other two groups, were higher, equivalent, and lower, respectively. **B** Principal component analysis of microglial morphology was performed using IBM SPSS Statistics software with 3000 microglia randomly sampled from each group. The observable core cluster of brain microglia in wild-type control group (black dots) is shown by an ellipse (~ 90%). The remaining cells were scattered either along the positive axis of principal component 1 (~ 6%) or along the negative axis of principal component 2 (~ 4%). Treatment with LPS (blue dots) increased the cells scattered out of the core to ~ 17%, which was further increased in LPS-treated Siglec-E knockout mice (red dots) to ~ 25%

We then carried out the morphological analyses on Iba1^+^ brain microglia using 26 measurements by NIH ImageJ/Fiji software (Table 1, https://imagej.nih.gov/ij/docs/menus/analyze.html) [49], and the normalized outcomes were shown in a radar chart (Fig. 3A). In addition, the principal component analyses were performed to illustrate the morphological changes of activated microglia upon the LPS treatment and deletion of Siglec-E (Fig. 3B). It appeared that the LPS-driven morphological alterations of microglia were largely augmented by the absence of functional Siglec-E (Fig. 3A, B). These findings together suggest that inducible Siglec-E plays an essential role in the regulation of microglial activation and that its depletion would exacerbate microgliosis within the brain exposed to PAMPs.

**Table 1:** The 26 measurements that are used to characterize the microglial morphology

Measurement	Definition
Kurtosis	The fourth-order moment about the mean
Min. gray level	Minimum gray values within the selection
Median	The median value of the pixels in the image or selection
Modal gray value	Most frequently occurring gray value within the selection
Mean gray value	Average gray value within the selection
Circularity	= 4π × area/perimeter^2^
Solidity	= Area/convex area
Max. gray level	Maximum gray values within the selection
Roundness	= 4 × area/(π × major_axis^2^), or the inverse of the aspect ratio
Average branch length	The average length of branches, in the corresponding units
Aspect ratio	= major_axis/minor_axis
Standard deviation	Standard deviation of the gray values
Maximum branch length	The maximum length of branches, in the corresponding units
MinFeret	The minimum caliper diameter
Feret’s diameter	The longest distance between any two points along the selection boundary
Integrated density	The product of area and mean gray value
Perimeter	The length of the outside boundary of the selection
End-point pixels	Count of pixels if they have less than 2 neighbors
Area	Area of selection in square pixels
Skewness	The third order moment about the mean
Branches	The number of branches
Slab pixels	Count of pixels if they have exactly 2 neighbors
Quadruple points	The number of triple points
Triple points	The number of quadruple points
Junctions	The number of actual junctions
Junction pixels	Count of pixels if they have more than 2 neighbors

More detailed information about these measurements can be found from https://imagej.nih.gov/ij/docs/menus/analyze.html and [49]

**Fig. 4:**
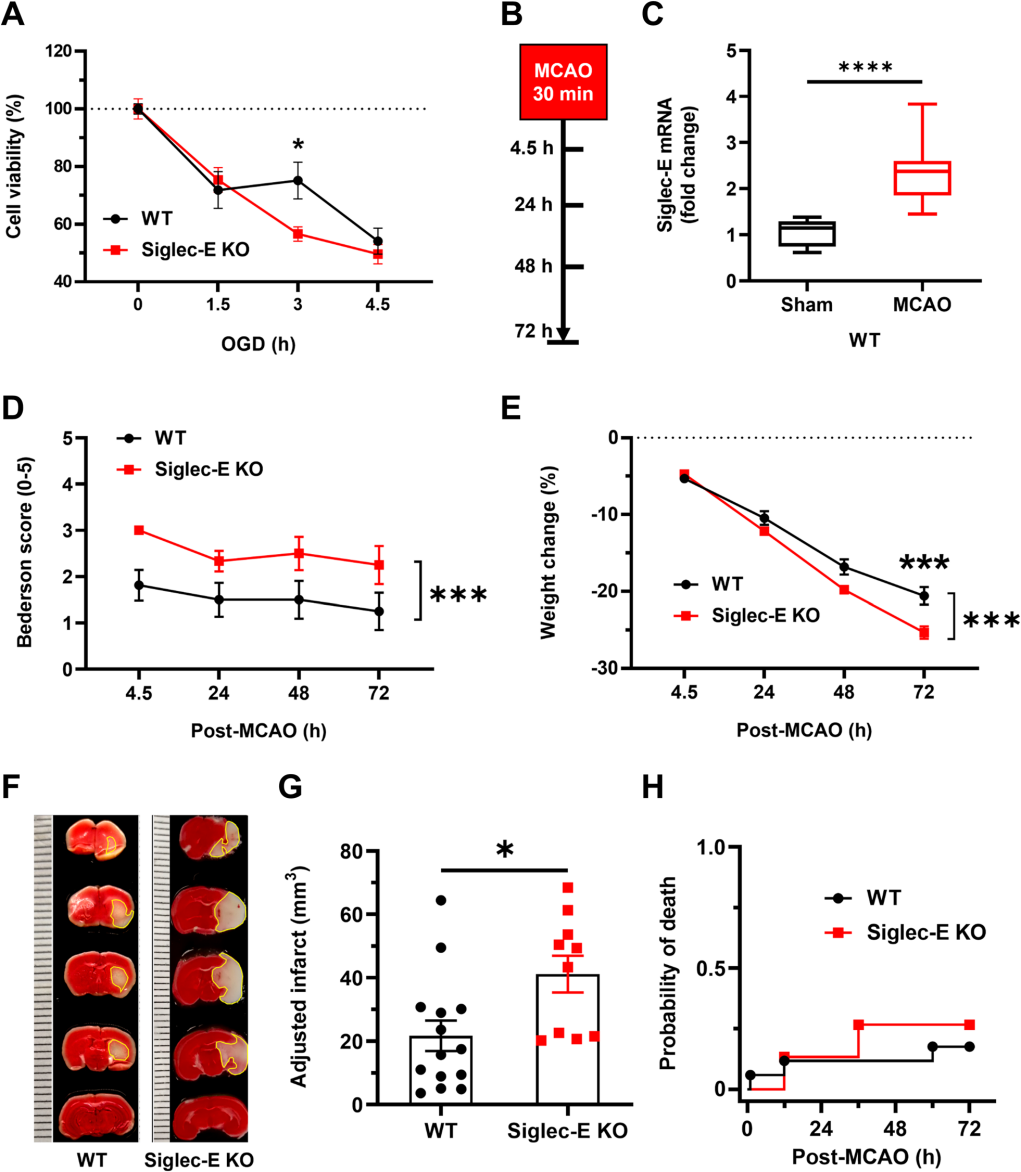
Induced Siglec-E is neuroprotective after ischemia–reperfusion injuries. **A** Primary neuron–glia cultures derived from cortices of wild type or Siglec-E knockout mouse embryos were subjected to oxygen–glucose deprivation (OGD) for 1.5, 3, or 4.5 h. Following reoxygenation with full nutrition supply for 16 h, the cell viability in these cultures was measured and compared (*n* = 8–24, *p = 0.0172, two-way ANOVA with post hoc Šidák multiple comparisons). Data are presented as mean ± SEM. **B** Intraluminal filament-based middle cerebral artery occlusion (MCAO) model was utilized to examine the effects of genetic ablation of Siglec-E on cerebral ischemia. In this study, adult wild type and Siglec-E knockout male mice (12–14 weeks old) were subjected to transient MCAO for 30 min, which was followed by reperfusion for 72 h. **C** Siglec-E mRNA expression in the ipsilateral brain tissues of mice subjected to 30-min MCAO and 72-h reperfusion was measured by qPCR and compared to that of sham cohort (*n* = 7–14, ****p < 0.0001, Mann–Whitney *U* test). Data are visualized using box plot. **D** Neurological deficits of wild type and Siglec-E knockout mice after MCAO were evaluated at multiple time points using Bederson’s scale (*n* = 12–16, ***p < 0.001, two-way ANOVA). Data are presented as mean ± SEM. **E** Deficiency of Siglec-E in mice exacerbated post-stroke weight loss (*n* = 12–16, ***p < 0.001, two-way ANOVA with post hoc Šidák multiple comparisons). Data are presented as mean ± SEM. **F** Triphenyltetrazolium chloride (TTC) staining was performed to measure the brain infarction in wild type and Siglec-E knockout mice 72 h after MCAO. Representative images from each cohort are shown. The viable brain parenchyma appeared reddish, whereas the infarcted areas were pale and highlighted. **G** The volumes of brain infarcts in wild type and Siglec-E knockout mice were quantified and compared (*n* = 10–14, *p = 0.022, Mann–Whitney *U* test). Data are presented as mean ± SEM. **H** Animal mortality over 72 h following transient MCAO for 30 min (*n* = 14–17, *p* = 0.5734, log-rank test)

In addition to neuroinflammation triggered by various PAMPs, sterile injured brain tissues can release DAMPs to activate innate immune receptors particularly on microglia, which, in turn, drive inflammatory reactions in neurological conditions such as ischemic stroke [38]. The induction of Siglec-E expression in the brain following ischemic stroke was first reported in a recent transcriptome screening study and appeared dependent on GPR68, a neuronal metabotropic proton receptor that mediates neuroprotection in acidotic and ischemic conditions [50]. We thus hypothesize that the inducible Siglec-E might be involved in an intrinsic neuroprotective strategy of the brain following ischemic injury. To test this hypothesis, we first examined the effects of genetic ablation of Siglec-E on oxygen–glucose deprivation (OGD)-induced cell death in mouse primary cortical cultures containing both neurons and glial cells. Primary cortical cultures from wild type or Siglec-E knockout mouse embryos (E18) were exposed to OGD for 1.5, 3, and 4.5 h, followed by reoxygenation and complete nutrition supplement for 16 h. The cell viability was measured using MTT assay, and we found that OGD induced cell death in these mouse primary cortical cultures in a time-dependent manner. Intriguingly, the neuronal death caused by moderate (3 h) but not mild (1.5 h) or severe (4.5 h) OGD was significantly augmented in neuro-glia mixed cortical cultures derived from Siglec-E knockout mice when compared to wild-type control cells (Fig. 4A).

Motivated by the promising neuroprotection of Siglec-E after moderate OGD, we next sought to determine the role of Siglec-E in ischemic injury in our mouse model of transient brain ischemia, in which middle cerebral artery occlusion (MCAO) lasts for about 30 min and is followed by reperfusion for 72 h (Fig. 4B). We first validated the significant induction of Siglec-E expression within the ipsilateral hemisphere of wild-type mice by a 30-min episode of MCAO when compared to the sham control (Fig. 4C), suggesting that a moderate transient brain ischemia was able to induce Siglec-E in the ischemic brain regions. We then found that the genetic ablation of Siglec-E led to more severe neurological deficits measured by Bederson scale during the 72-h reperfusion (Fig. 4D). Likewise, the Siglec-E knockout mice overall experienced higher post-stroke weight loss when compared to the wild-type control animals (Fig. 4E). Intriguingly, the neurological deficits of both wild type and Siglec-E knockout mice appeared to stabilize since 24 h after MCAO (Fig. 4D), whereas their weight loss continued throughout the experimental period (Fig. 4E). The observation that more weight loss was not necessarily associated with severer neurological deficits suggests that the assessment of neurological deficits by Bederson scale was unlikely interfered by the concomitant weight loss.

A 30-min episode of MCAO was sufficient to cause moderate brain damages in wild-type mice, indicated by medium-sized brain infarcts (~ 20 mm^3^) found in the cortex and striatum (Fig. 4F). In line with the worsened post-stroke wellbeing in Siglec-E knockout mice, the MCAO-triggered brain damages were exacerbated by the genetic deletion of Siglec-E, as the brain infarct volumes were nearly doubled in Siglec-E knockout mice when compared to the wild-type cohort (Fig. 4G). However, the ablation of Siglec-E did not significantly alter the post-stroke mortality rates in mice (Fig. 4H). Nonetheless, these post-stroke outcomes together support a neuroprotective role of induced Siglec-E following ischemic stroke.

## Discussion

Activation of Siglec-E by sialic acid-containing glycans has been increasingly recognized as a critical immunosuppressive mechanism in response to various PAMPs and DAMPs. The expression of Siglec-E is low in the naïve brain, but with inflammatory or injurious stimulation it is rapidly and robustly induced, particularly in microglia. In this study, we found that the genetic ablation of Siglec-E led to a vigorous increase of the LPS response of microglia both in vitro and in vivo. The Siglec-E deficiency also aggravated OGD-triggered neuronal death in mouse primary cortical neuron–glia mixed cultures and augmented ischemic brain injury in mice. These new findings demonstrate profound anti-inflammatory and neuroprotective functions of induced Siglec-E following exposure of the brain to PAMPs or DAMPs. As such, the current study provides the *first* indication of an intrinsic self-protection mechanism of the brain for inflammation resolution and tissue repair after ischemic stroke or other acute neurological insults.

The mouse ENCODE transcriptome data (https://www.ncbi.nlm.nih.gov/gene/83382) suggest that Siglec-E is most abundantly expressed in the adult spleen [51]. In the CNS, the Allen Mouse Brain Atlas has reported that the expression of Siglec-E is mainly enriched in olfactory bulb, hippocampus, neocortical layer II, and cerebellum (http://mouse.brain-map.org/gene/show/57631). Although the basal expression of Siglec-E in the brain is overall low, it can be rapidly and robustly induced by various injurious and inflammatory stimuli. In this study, we found that Siglec-E was markedly upregulated under both PAMPs and DAMPs-related disease conditions. In particular, the upregulated Siglec-E showed colocalization with microglial marker Iba1 in the brain after LPS treatment in mice, suggesting that microglia are a major cellular source for Siglec-E expression in brain upon challenges. Intriguingly, the elevated Siglec-E appeared to diminish the surge of proinflammatory mediators produced by microglia, reduce the number of microglia, and counteract their morphological changes when they are activated. As such, Siglec-E likely plays a preventive role in the microglial transformation from resting state to activated state. However, it should be noted that an evident portion of the Siglec-E was detected in cells that did not express Iba1 (Iba-1^−^), indicating that microglia may not be the only type of cells expressing Siglec-E in the brain responding to inflammatory challenges. Likewise, microglia may not be the only brain-resident immunoreactive cells expressing Siglec-E upon LPS stimulation or ischemic injury, as peripheral Iba-1^+^ myeloid cells can infiltrate into the brain parenchyma under these conditions. Future investigation using cell type-specific knockout strategies is needed to identify all these Siglec-E-expressing cells (Iba-1^+^ or Iba-1^−^) in the brain in order to fully understand the roles of Siglec-E in health and disease.

Excessive reactive microgliosis is sufficient to cause neuronal damage and is considered pro-neurodegenerative, as the activation of some pattern recognition receptors expressed on the microglial surface often leads to the release of reactive oxygen species (ROS) that can trigger neurotoxicity [52]. Conversely, as another type of pattern recognition receptors expressed by microglia, Siglec-E overall is immunosuppressive, and it functions against reactive microgliosis. Previous evidence supports the notion that microglial Siglec-E is neuroprotective through attenuating neuroinflammation and phagocytosis-associated oxidative stress [33, 53]. Microglial Siglec-E was shown to prevent neurotoxicity through interacting with the sialic acid decorated on the neuronal glycocalyx [53]. In line with this, our findings support anti-inflammatory and neuroprotective roles of Siglec-E after ischemic stroke. Thus, pharmacological activation of Siglec-E may trigger the ITIM-mediated intracellular immunoinhibitory signaling and predispose activated myeloid immunocytes to a quiescent state. Future studies should also be directed to identify therapeutic agents that can directly activate Siglec-E. For instance, pS9L-lipid, a glycopolypeptide ligand as a *cis*-binding agonist for Siglec-E and Siglec-9 (the human ortholog of mouse Siglec-E), can inhibit TLR4-induced NF-κB activity, MAPK signaling, and phagocytosis by macrophages and microglia [54], demonstrating *cis*-binding agonists as a way to activate Siglecs in inflammatory diseases. Whether these *cis* ligands can cross the blood–brain barrier and be useful to suppress post-stroke neuroinflammation following brain insults such as ischemic stroke is another interesting question that remains to be answered. Similarly, selective sialidase inhibitors, which aim to increase the Siglec-E activity and suppress inflammatory response [44, 55], should also be tested in animal models of MCAO for potential use in stroke treatment.

In sum, our findings indicate that the Siglec-E in microglia is rapidly induced by LPS and ischemic stroke and, in turn, leads to beneficial, anti-inflammatory, and neuroprotective effects under these neuroinflammatory conditions. This suggests that Siglec-E activation by selective agonists might represent a novel immunomodulatory strategy to confer neuroprotection in cerebral ischemia and other acute or chronic neurological conditions in which neuroinflammation facilitates neuronal death.

## Data Availability

All relevant data in this study are available upon reasonable request directed to the corresponding author.

## Abbreviations

DAMPs: Damage-associated molecular patterns
DAP12: DNAX-activating protein of 12 kDa
ITAM: Immunoreceptor tyrosine-based activation motif
ITIM: Immunoreceptor tyrosine-based inhibitory motif
KARAP: Killer cell activating receptor-associated protein
LPS: Lipopolysaccharide
MAPK: Mitogen-activated protein kinase
MCAO: Middle cerebral artery occlusion
NF-κB: Nuclear factor κB
OGD: Oxygen–glucose deprivation
PAMPs: Pathogen-associated molecular patterns
ROS: Reactive oxygen species
Siglec-E: Sialic acid immunoglobulin-like lectin E
TBI: Traumatic brain injury
TLR4: Toll-like receptor 4
TYROBP: Tyrosine kinases binding protein
